# Perceptions of Hansen's Disease in Northeast Brazil: A Community‐Based Study Integrating Stigma, Empowerment, and Social Distance

**DOI:** 10.1111/tmi.70087

**Published:** 2026-02-26

**Authors:** Jaqueline Caracas Barbosa, Aymée Medeiros da Rocha, Hellen Xavier Oliveira, Nágila Nathaly Lima Ferreira, Adriana da Silva dos Reis, Anderson Fuentes Ferreira, Wim H. van Brakel, Anna T. van ‘t Noordende, Duane Charles Hinders, José Alexandre Menezes da Silva, Carmem Emmanuely Leitão Araújo, Rômulo do Nascimento Rocha, Alberto Novaes Ramos

**Affiliations:** ^1^ Postgraduate Program in Public Health, Faculty of Medicine Federal University of Ceará Fortaleza Brazil; ^2^ NHR Brasil Fortaleza Brazil; ^3^ Instituto Nacional de Infectologia Evandro Chagas Fiocruz‐Rio de Janeiro Rio de Janeiro Brazil; ^4^ Department of Public Health Emergencies Ministry of Health, Secretariat for Health and Environmental Surveillance Brasília Brazil; ^5^ NLR Amsterdam the Netherlands; ^6^ Department of Community Health, Faculty of Medicine Federal University of Ceará Fortaleza Brazil

**Keywords:** empowerment, Hansen's disease, knowledge, social stigma

## Abstract

Perceptions of Hansen’s disease (HD) can influence the level of stigma, empowerment, and intention to distance oneself from those affected by the disease. Knowledge, attitudes, and beliefs shape these perceptions. This study aims to understand perceptions of HD regarding stigma and empowerment. Method: A cross‐sectional, mixed‐methods research design was employed in endemic communities in the State of Ceará. The instruments were applied to people affected by HD, their contacts, community members, and health workers (including nurses, doctors, dentists, psychologists, occupational therapists, and others), as well as Community Health Agents (CHAs). The study assessed socio‐demographic status, beliefs, knowledge, attitudes, and practices (KAP). Individual stigma was measured using the Explanatory Model Interview Catalogue – EMIC‐AP; community stigma using the EMIC Community Stigma Scale – EMIC‐CSS; social distance using the Social Distance Scale – SDS; and empowerment using the Empowerment Scale – ES. Semi‐structured interviews and five focus group discussions were also conducted. Quantitative data were analyzed using descriptive statistics and multivariate regression. Qualitative data were analyzed using thematic analysis. Findings: A total of 1,309 participants were included in the study: 203 people affected, 251 contacts, 350 community members, 302 CHAs, and 203 high‐level health workers. A total of 89 qualitative interviews (in both municipalities) and five focus groups (in each municipality) were conducted. Items relating to knowledge of the cause, transmission, and duration of the disease were the lowest‐scoring items, especially among community members, who also had the lowest average HD knowledge score and the highest average score on the social distancing scale. Community Health Agents (CHAs) reported more stigmatizing attitudes in the EMIC‐CSS than community members did. Those affected perceived a high level of stigma and had low levels of empowerment. Conclusion: The community had poor knowledge of HD and exhibited negative attitudes towards affected individuals; stigma is still present today. This study highlighted the need for community health education and ongoing education from healthcare professionals. Health education plays an essential role in HD. There is also a need for strategies to overcome stigma and prejudice, and to improve understanding of the disease beyond its biological aspects.

## 1 Introduction

Hansen's disease (HD), also known as leprosy, is a chronic infectious disease of the skin and peripheral nerves caused by *Mycobacterium leprae* , which can affect sensory, motor, and autonomic nerve functions and result in physical disability [1, 2]. In 2023, Brazil had the second‐highest number of new HD cases worldwide and the highest in the Americas, with 22,773 cases diagnosed [2].

Following the introduction of multidrug therapy, the number of HD cases fell dramatically, then declined more slowly, and then gradually decreased after 2000. However, transmission has persisted, including in Brazil. Predictive studies indicate that new tools are needed to interrupt HD transmission, such as chemoprophylaxis [3]. To achieve a 90% reduction in the next 22 years, preventive treatment must be offered to 40.2 million contacts [3, 4, 5, 6, 7]. For such new technologies to be successfully implemented, people affected by HD, their contacts, and communities need to recognise the disease, understand its treatment and prevention options, and feel able to seek and offer care. Historically, HD has been a stigmatised disease, shrouded in various beliefs, prejudices, and discrimination. Despite having existed for thousands of years, knowledge about HD, which remains limited in many settings, and negative attitudes, such as labelling, stereotyping, and discrimination, persist. Stigma has multiple dimensions and interferes with the personal and social lives of people affected by HD. It also has psychological and financial impacts and restricts social life [4, 5, 6, 7].

Stigma compromises not only the timely diagnosis and treatment of HD, but also other strategic public health interventions, such as BCG vaccination and chemoprophylaxis for contacts. For this reason, the Brazilian Ministry of Health's recent clinical and therapeutic guidelines recommended using stigma and social participation scales to evaluate the psychosocial effects of HD and to inform strategies to address stigma and social participation restrictions [1].

Assessments of knowledge, beliefs, attitudes, and practices relating to HD have been widely conducted and reported in the international literature [8, 9, 10, 11, 12]. In general, these studies reveal a lack of knowledge about HD, as well as stigmatising beliefs and negative attitudes towards those affected [4, 9, 10, 12]. Notably, several studies have employed expanded mixed‐method approaches to analyse different population groups within the same area and to link findings to the local healthcare network [9, 10].

There is a compelling need to deepen understanding of HD beyond its biological aspects, incorporating perceptions, stigma, and social representations of the disease. Analysing these dimensions provides the basis for more effective intervention targeting, improving knowledge and perception of HD, and reducing the associated stigma [13, 14, 15]. However, relatively few studies with such a focus have been conducted in Brazil, despite the high disease burden [16, 17, 18].

In this context, stigma can be understood as the result of stereotyping, labelling, and discrimination against people with HD, often expressed as rejection and exclusion in families, communities, institutions, and organisations [5]. Therefore, there is an increasing need for studies that examine perceptions of HD, as these perceptions can influence stigma, empowerment, and social distancing among those affected. Here, social distance refers to the desire to avoid contact or relationships with people affected by HD in everyday situations [19].

Another key concept in this study is “empowerment”, which we understand as a process through which individuals and groups increase their control over decisions and actions that affect their lives, moving from a position of dependence and powerlessness to one of agency and collective action [11, 20, 21].

In Brazil, the term is often used to refer to both mobilisations and practices that encourage groups and communities to improve their living conditions, as well as actions that promote the inclusion of socially vulnerable populations, with the common aim of expanding autonomy and participation [20, 21].

The present study, therefore, aims to assess perceptions of HD, including knowledge, attitudes, beliefs, and practices, across different study populations in specific endemic areas of the Northeast region of Brazil. We analyse not only prior knowledge but also stigma, empowerment, and desired social distance to inform more effective strategies that guarantee comprehensive and equitable access to healthcare and strengthen HD control.

## 2 Methods

### 2.1 Study Design

This community‐based study employed a cross‐sectional design and used mixed methods. It represents a strategic baseline study integrated into the PEP++ project in Brazil: a pragmatic, cluster‐randomised controlled clinical trial of enhanced post‐exposure chemoprophylaxis for contacts of people affected by HD in the cities of Fortaleza and Sobral, in the state of Ceará [22].

### 2.2 Study Area

**FIGURE 1:**
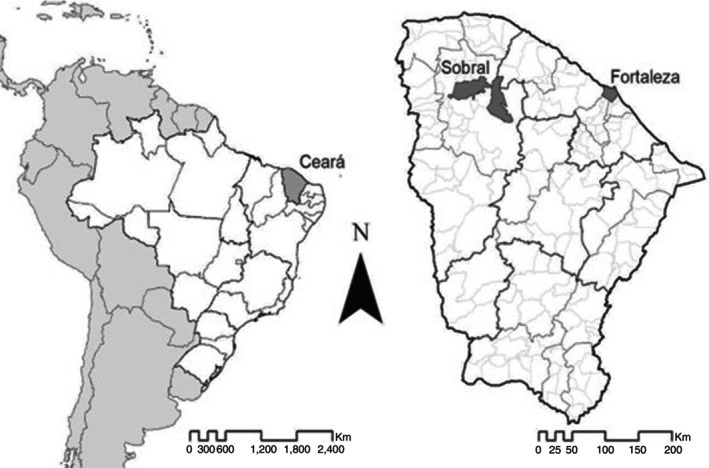
Study areas: State of Ceará, Municipalities of Fortaleza and Sobral, Brazil.

The research was conducted in the cities of Fortaleza and Sobral, in the state of Ceará, northeastern Brazil, and was included in the PEP++ study (see Figure 1).

In Fortaleza, the capital of the state of Ceará, areas of high and medium HD case concentration were identified by the municipal HD control programme using routinely collected data from the national notifiable diseases information system. Primary health centre catchment areas were classified into strata based on their mean annual HD case detection rates in the years preceding the study, and primary health centres (PHCs) located in high‐ and medium‐concentration strata were eligible for inclusion. Three health regions and eight PHCs were selected. In Sobral, fieldwork was conducted across the urban and part of the rural areas, covering 22 PHCs.

### 2.3 Study Population and Sampling

The following subpopulations were considered in the study areas: (a) Persons affected by HD (“index patients”)—diagnosed and reported in 2017 and 2018 and who were residing in the municipality of Sobral and the primary health centre catchment areas of Fortaleza classified as high‐ or middle‐endemic for HD, as described above; (b) Household and social contacts—family or non‐family members listed by the index patients as close contacts, with whom they interacted closely and for a prolonged period of time (for approximately 20 h per week for at least three months in the year preceding the index patient's diagnosis), including household members, neighbours, close friends, co‐workers and classmates; (c) Community members—residents of the study area at the time of the survey who were not reported as household or social contacts; (d) Community Health Agents (CHAs) and other healthcare workers, including doctors, nurses, dentists, physiotherapists, occupational therapists, psychologists, and speech therapists, who were part of the PHC teams. CHAs were considered as a distinct group from other healthcare workers because they are lay health workers who usually live in the communities where they work, have different training and socio‐demographic profiles, and play a specific role in household‐level outreach and HD control.

The following individuals were excluded: those under 18 years of age; those unable to participate in the planned research activities due to cognitive or neurological impairments that limit understanding and interaction; and those unable to give informed consent. Contacts, community members, and healthcare workers (in these cases, professionals who had worked in the PHC for at least 6 months) who had also been diagnosed with HD at any time were not included in the study.

The quantitative component of the study used a sampling method that selected all health workers who were part of the PHC teams and were present at the time of the research, as well as those who were eligible for the study (having worked in the area for more than six months and not having received an HD diagnosis).

It was estimated that in each municipality, 25 doctors, 25 nurses, 25 dentists, and 25 other professionals (such as dentists, physiotherapists, occupational therapists, psychologists, and speech therapists) would be approached, as well as 150 CHAs.

The study included people who were diagnosed with HD between 2017 and 2018. For each index patient included in the study, their contacts were listed. It was initially planned to approach one household contact per index patient, with a maximum of 30 social contacts per index patient.

To select the subpopulation of community members, we first selected 50 people affected by HD in each municipality, based on the proximity of their homes to the health unit. Taking each patient as a reference point, seven community members were selected through convenience sampling from different houses. This involved considering 10 neighbouring houses to the right and left of each patient's home, for a total of 175 people in each municipality.

An assumed 50% ‘negative attitudes’ rate was used as a baseline before any intervention, with a reduction of at least 15% after the intervention considered the minimum detectable difference. Based on these parameters and a significance level of 0.05, the study had 80% power to detect such differences.

The exact baseline prevalence was used as a reference to calculate the following samples: 100 index patients and 100 households, and 130 social contacts, before and after this intervention, to detect a reduction in negative attitudes of 20% or more.

As for the qualitative component of the study, the initial plan was to select participants using purposive sampling, with six people from each subpopulation (three female and three male) and five focus groups in Sobral and five in Fortaleza. In practice, 89 individual interviews and ten focus group discussions were conducted across the two municipalities (Table 1). The number of interviews per subpopulation was allowed to vary to achieve thematic saturation and capture the diversity of experiences, particularly across different categories of healthcare workers and CHAs.

**TABLE 1:** Number of participants included in the study by subpopulation in the cities of Fortaleza and Sobral, 2020.

Subpopulations	Questionnaires (%)	Interviews (%)	Focus group discussion (%)
Index patient	203 (15.5)	12 (13.5)	11 (13.9)
Close contact	251 (19.2)	18 (20.2)	10 (12.7)
Community member	350 (26.7)	17 (19.1)	7 (8.9)
Health care worker	203 (15.5)	30 (33.7)	21 (26.6)
Community Health Agent	302 (23.1)	12 (13.5)	30 (37.9)
**Total**	**1309 (100.0)**	**89 (100.0)**	**79 (100.0)**

Participation was encouraged through representative stories that showed the various perspectives on HD observed during data collection. Therefore, the qualitative sample was a subset from the quantitative component aimed to ensure equitable representation by age and sex/gender.

### 2.4 Data Collection

The data collection process was based on the selected PHC operating areas. Participants could choose to be interviewed at home, at the health unit, or in their workplace. All interviewers received training on HD, the instruments used, and interviewing techniques before data collection.

A pilot phase was conducted before data collection to allow minor adjustments to the interview guide. Participants in this phase were not included in the final sample. No changes were made to the questionnaires used. Data were collected between November 2018 and September 2019. All scales, KAP, EMIC‐AP, EMIC‐CSS, Empowerment scale, and SDS [18, 23, 24, 25, 26] were translated into Brazilian Portuguese and were also cross‐culturally adapted.

A total of six instruments were used to assess the quantitative components. Table 2 summarises how these instruments were applied across the subpopulations included in this study. It is worth noting that the income variable in the socio‐demographic instrument was used only to index patients' contacts, community members, and CHAs living in these areas (188 CHAs). Health workers were excluded because the aim was to understand the social and economic aspects of the study area, and most health workers do not live in the area where they work; only 10 of 203 health workers in this group lived in the study area. Only health workers and CHAs who lived in the area were interviewed with the SDS and EMIC instruments.

**TABLE 2:** Application of the instruments to the subpopulations of the CAPP‐HANS study.

Subpopulations	Sociodemographic	KAP	EMIC‐CSS Scale	SDS scale	EMIC‐AP Scale	Empowerment Scale
Persons affected by HD (index patients)	**X**	**X**	—	—	**X**	**X**
Contacts of persons affected by HD	**X**	**X**	**X**	**X**	—	—
Community members	**X**	**X**	**X**	**X**	—	—
Health workers^a^	**X**	**X**	**X**	**X**	—	—
Community Health Agents^a^	**X**	**X**	**X**	**X**	—	—

^a^ The EMIC‐CSS and SDS scales are only applied to those who reported living in the PHC areas.

### 2.5 Knowledge, Beliefs, Attitudes, and Practices

Data on knowledge, beliefs, attitudes, and practices (KAP) related to HD were obtained from each study sample using a 17‐item instrument with questions, ranging from 0 to 7 points consisting of single or multiple‐answer questions with no suggested answer options for respondents. For questions with multiple answer options, a response was considered correct only if it was given without indicating any incorrect answers. This instrument generates a KAP score by summing the seven items. This was defined as ‘low or poor knowledge’ (0–2 correct answers), ‘medium or moderate knowledge’ (3–4 correct answers), and ‘high or adequate knowledge’ (5–7 correct answers). This score was standardised in a study conducted in India [9] and has since been used in other studies conducted in India and Indonesia [10].

### 2.6 EMIC‐AP and EMIC‐CSS

Individual stigma in people affected by HD was assessed using the *Explanatory Model Interview Catalogue Stigma Scale* (EMIC‐AP). This scale comprises 15 specific items that generate a score ranging from zero (no perceived stigma or self‐stigma) to 45 (a high degree of both) [18, 24, 26, 27].

The *Explanatory Model Interview Catalogue Community Stigma Scale* (EMIC‐CSS) and the *Social Distancing Scale* (SDS) were administered to subpopulations, including household and social contacts, community members, CHAs, and health workers. The EMIC‐CSS scale was used only for healthcare workers who reported living in the PHC area where they worked.

The EMIC‐CSS instrument was used to measure attitudes and behaviours towards people affected by HD. It consists of 15 items, with a total score ranging from zero (no negative attitudes) to 30 (most negative attitudes).

### 2.7 Empowerment Scale

Empowerment was assessed using Rogers's Empowerment Scale [23]. The ES comprises 25 items divided into five concepts or domains: self‐esteem, autonomy, power, optimism, and righteous anger. The ES scores range from 0 (no manifestation of empowerment) to 100 (highest level of empowerment) [23, 26].

For this paper, these domains are defined as follows: Self‐esteem—a set of feelings and thoughts about one's own worth, competence, and adequacy, which are reflected in a positive or negative attitude; Autonomy—self‐government; Power—the capacity or authority to do something; Optimism—defined as the tendency to view things positively and think positively. Optimistic: people have positive expectations of success and achievement, even when they face significant difficulties or failures. Righteous anger: anger justified by the provocation suffered. It is considered a primary emotion and is functionally necessary for the survival of the species [23, 26].

### 2.8 SDS

The SDS measures the desired social distance a person interviewed wishes to maintain towards someone with a specific condition, such as HD. The *Social Distance Scale* (SDS) is an internationally validated seven‐item scale that assesses the social distance the respondent wishes to maintain from a person described in a vignette. The social distance score reflects the respondent's attitudes and fears, producing a sum score ranging from 0 (no desired social distance, no fear, or negative attitudes) to 21 (maximum desired social distance, lots of fear, and negative attitudes) [19, 25].

### 2.9 Qualitative Component

The qualitative component of the study, comprising individual interviews and focus groups, underwent thematic analysis of the interview data. This analysis comprised three stages: pre‐analysis, exploration of the material or coding, and treatment of the obtained results/interpretation [28]. The transcripts in this study were anonymized and grouped by study subpopulation. These transcripts were then read and re‐read to gain an overall perspective and to identify similarities and differences. Data collection for the qualitative component continued in each subpopulation until thematic saturation was reached, that is, no new relevant themes emerged in subsequent interviews or focus groups.

### 2.10 Data Analysis

The sociodemographic data obtained from the research instruments (KAP, EMIC‐AP, ES, EMIC‐CSS, and SDS) and the study populations were described using relative and straightforward frequencies or mean scores (domain‐specific and/or total scores, where applicable), alongside their respective 95% confidence intervals (95% CI).

Multivariate linear regression analysis was employed. Binary variables were created from recategorized variables with multiple categories. The independent variables in the model were age, sex, subpopulation, education, occupation, whether the respondent knew someone affected by HD, and community, while knowledge, stigma, empowerment, and social distance were treated as dependent variables.

The regression coefficients were interpreted as ranging from negative to positive values; negative coefficients indicated an inverse relationship with the variables of interest and a reduced probability of the analysed outcome. In addition to the regression coefficients, the results reported 95% confidence intervals (CI) and *p*‐values.

Variables with *p*‐values < 0.20 in the univariate analysis were included in the multivariate model, whereas those showing collinearity were excluded. Non‐normality of the outcome variables was addressed using bootstrapping, without assuming a modal distribution.

The multivariate analysis was conducted using stepwise backward elimination. Three models were developed: (a) a complete dataset model (including all municipalities and subpopulations), (b) a model focusing on healthcare professionals (including Community Health Agents—CHAs), and (c) a model including individuals with the disease, their contacts, and members of the community.

For the qualitative component, thematic analysis was employed in interviews, and focus group discussions were conducted with specific target groups. Participants' identities were anonymized in the transcripts. The transcripts were organized to provide a comprehensive overview.

The quantitative data derived from the field interviews were organised and consolidated using EpiInfo 7 (US Centers for Disease Control and Prevention—CDC, Atlanta, GA). Descriptive and analytical statistical analyses were then performed using Stata Statistical Software, Release 11 (StataCorp LP, College Station, TX).

### 2.11 Ethical Aspects

The CAPP‐HANS project was first submitted to the Research Ethics Committee of the Federal University of Ceará (UFC) and later, as part of an international multicenter project, submitted to the National Research Ethics Committee (CONEP) of Brazil. CONEP is directly linked to the National Health Council (CNS). Approval was granted under the Certificate of Ethical Assessment Presentation (CAAE) No. 86480218900005054 and approval No. 2986692. All current CNS resolutions (466/2012, 510/2016, and 292/1999) were adhered to. All participants were informed of the study's objectives and procedures, as well as the conditions for the secrecy and confidentiality of the data, before data collection. Written informed consent to participate in the study was obtained from each participant.

## 3 Results

A total of 1309 participants were involved, including 203 (15.5%) new HD cases, 251 (19.2%) contacts, 350 (26.7%) community members, 203 (15.5%) healthcare workers, and 302 (23.1%) CHAs. In addition to the quantitative component of the study, 89 individual interviews and ten focus group discussions were conducted with 79 individuals from the various participating sub‐populations (see Table 1). These qualitative participants were distributed across the different subpopulations, with a higher number of interviews among healthcare workers and CHAs to reflect the heterogeneity of professional categories and workplaces (Table 1).

Of those evaluated, 664 (50.7%) were from Fortaleza and 645 (49.3%) were from Sobral. Most were female (*n* = 934, 71.4%), married (*n* = 599, 45.8%), and Catholic (*n* = 840, 64.2%). The overall mean age was 43.5 years (95% CI 42.7 to 44.3). Among the subpopulation of people affected by HD, their contacts, and community members, 381 (29.1%) had completed elementary education, 101 (7.7%) were illiterate, and 299 (22.8%) reported having an income of less than one [1] minimum wage. Table 3 provides an overview of the study subpopulations by sociodemographic characteristics.

**TABLE 3:** Characterisation of the participants included in the study by subpopulations and socio‐demographic aspects in the cities of Fortaleza and Sobral, 2020.

Variables	Subpopulations					
	Index patient	Close contact	Community member	Health worker	Community Health Agent^a^	Total
**Total sample (row %)**	**203 (15.5)**	**251 (19.2)**	**350 (26.7)**	**203 (15.5)**	**302 (23.1)**	**1,309 (100.0)**
**Municipality of residence**						
Fortaleza	102 (50.2)	133 (54.0)	175 (50.0)	102 (50.2)	152 (50.3)	664 (50.7)
Sobral	101 (49.8)	118 (47.0)	175 (50.0)	101 (49.8)	150 (49.7)	645 (49.3)
**Sex**						
Male	110 (54.2)	73 (29.0)	95 (27.1)	48 (23.6)	49 (16.2)	375 (28.6)
Female	93 (45.8)	178 (70.9)	255 (72.8)	155 (76.3)	253 (83.8)	934 (71.4)
**Marital status**						
Married/living together	113 (55.7)	145 (57.8)	227 (64.9)	100 (49.3)	187 (61.9)	772 (59.0)
Others (single, separated/divorced/widower)	90 (44.3)	106 (42.2)	123 (35.1)	103 (50.7)	115 (38.1)	537 (41.0)
**Religion**						
Catholic	143 (70.4)	148 (59.0)	203 (58.0)	160 (78.8)	186 (61.6)	840 (64.2)
Other (evangelical, spiritism, afro‐Brazilian)	41 (20.2)	103 (41.0)	107 (42.0)	30 (14.8)	102 (33.8)	423 (32.3)
Without religion	19 (9.4)	0 (0.0)	0 (0.0)	13 (6.4)	14 (4.6)	46 (3.5)
**Mean age in years (Range)**	51.5 (18 to 92)	40.6 (18 to 83)	45.4 (18 to 87)	39.3 (24 to 69)	41.4 (20 to 71)	43.6 (18 to 92)
**Education**						
Illiterate	35 (17.2)	17 (6.8)	49 (14.0)	—	—	101 (7.7)
Elementary school	109 (53.7)	103 (41.0)	160 (45.7)	—	9 (3.0)	381 (29.1)
High school	56 (27.6)	113 (45.0)	131 (37.4)	—	184 (60.9)	484 (37.0)
University education	3 (1.5)	18 (7.2)	10 (2.9)	203 (100.0)	109 (36.1)	343 (26.2)
**Income in minimum wages (Except health workers)** ^b^						
No income	34 (16.7)	22 (8.8)	117 (33.4)	—	—	—
< 1 minimum wage	21 (10.3)	37 (14.7)	68 (19.4)	—	—	—
1 minimum wage	77 (37.9)	64 (25.5)	89 (25.4)	—	—	—
2 to 3 minimum wages	20 (9.9)	33 (13.1)	33 (9.4)	—	—	—
≥ 4 minimum wages	33 (16.3)	53 (21.1)	34 (9.7)	—	—	—
Missing data	18 (8.9)	42 (16.7)	9 (2.6)			

^a^ CHAs are considered in Brazil as health workers and is not required university education to work in the primary health care, for that reason in this analysis we consider two groups: healthcare workers (high level) and CHAs.

^b^ Minimum wage per month in Brazil, reference to January 2019, the amount was of R$998,00 (equivalent to about 259 dollars).

Of those who participated in the qualitative interviews, 34 (53.1%) were women, with a mean age of 46.4 years (95% CI 45.6 to 47.8). In the focus group, 45 (56.9%) were women, with a mean age of 47.8 years (95% CI 46.7 to 48.7).

Table 4 provides an overview of the responses to the KAP assessment regarding HD knowledge. Overall, respondents most frequently reported ‘skin patches’ as the signs most associated with the disease, with 1002 responses (76.5%), followed by ‘loss of sensation’ (415 responses, 31.7%). Notably, in the CHA group, only 19 (5.6%) respondents associated the disease with loss of sensation. In the community, 135 people (38.6%) were unaware of the signs and symptoms.

No … I don't know why … as I've just told you … there are a lot of people who don't even know what it is […]. (community member).

**TABLE 4:** An overview of the responses given per knowledge question.

Variables	Subpopulations					
	Index patient	Close contact	Community	Health care workers	Community health agent	Participants with correct answers
**Total**	**203**	**251**	**350**	**203**	**302**	**1309**
**Early symptoms**						
*Skin patches* ^a^	152 (74.9)	188 (74.9)	192 (54.8)	183 (90.1)	287 (95.0)	*981 (74.9)*
*Loss of sensation* ^a^	88 (43.3)	77 (30.7)	78 (22.3)	153 (75.4)	19 (5.6)	
Don't know	12 (5.9)	34 (13.4)	135 (38.6)	0 (0.0)	1 (0.3)	
Itching	23 (11.3)	18 (7.1)	21 (8.4)	4 (2.0)	6 (2.0)	
Others: tingling, cough, bleeding, blisters, rashes	51 (25.1)	38 (15.1)	27 (7.7)	46 (22.7)	78 (25.8)	
**Cause of HD**						
Don't know	168 (82.7)	182 (72.5)	295 (84.3)	22 (10.8)	105 (34.8)	
*Germs/Bacteria* ^a^	24 (11.8)	27 (10.7)	17 (4.6)	142 (69.9)	84 (27.8)	*294 (22.5)*
Unclean environment	3 (1.5)	9 (3.6)	4 (1.1)	5 (2.5)	19 (6.3)	
Others: punishment for sins, karma, impure blood, hereditary	8 (3.9)	26 (10.3)	22 (6.3)	87 (42.8)	133 (44.0)	
**Transmission of HD**						
Don't know	102 (50.2)	116 (46.2)	246 (70.3)	3 (1.5)	17 (5.6)	
Skin contact	12 (5.9)	22 (8.8)	13 (3.7)	7 (3.4)	16 (5.3)	
Eating together	1 (0.4)	1 (0.4)	3 (0.8)	0 (0.0)	0 (0.0)	
Others: contaminated soil, insects, ‘different’	30 (1.5)	27 (10.7)	28 (8)	56 (27.6)	69 (22.8)	
*By air* ^a^	61 (30.0)	64 (25.5)	26 (7.4)	98 (48.2)	185 (61.2)	*434 (33.2)*
**Treatability of HD**						
*Can be treated* ^a^	200 (98.5)	245 (97.6)	318 (90.8)	203 (100)	300 (99.4)	*1266 (96.7)*
Don't know	1 (0.4)	4 (1.6)	6 (1.7)	0 (0)	1 (0.3)	
Can't be treated	2 (1.0)	2 (0.8)	26 (7.4)	0 (0)	1 (0.3)	
**How is it treated?**						
*Medication* ^a^	200 (98.5)	239 (95.2)	264 (75.4)	200 (98.5)	298 (98.7)	*1201 (91.8)*
Other treatment	3 (1.5)	12 (4.8)	86 (24.6)	3 (1.5)	4 (1.3)	
**Contagiousness**						
*Not contagious when on treatment* ^a^	163 (80.2)	222 (88.4)	291 (83.1)	200 (98.5)	285 (94.3)	*1161 (88.7)*
Infectious when on treatment	17 (8.4)	29 (11.5)	59 (16.8)	3 (1.5)	17 (5.6)	
Don't know	23 (11.3)	0 (0.0)	0 (0.0)	0 (0.0)	0 (0.0)	
**Disabilities**						
*Disabilities can be prevented* ^a^	160 (78.8)	212 (84.5)	266 (85.4)	198 (97.5)	286 (94.7)	*1122 (85.7)*
Don't know	25 (12.3)	0 (0.0)	0 (0.0)	0 (0.0)	0 (0.0)	
Disabilities can't be prevented	18 (8.9)	39 (15.5)	84 (14.6)	5 (2.5)	16 (5.3)	
**Duration of disease**						
*HD is temporary* ^a^	128 (63.0)	182 (72.5)	190 (54.3)	159 (78.3)	231 (76.5)	*890 (68.0)*
HD is permanent	46 (22.7)	51 (20.3)	99 (28.3)	40 (19.7)	61 (20.2)	
Don't know	29 (14.3)	18 (7.2)	61 (17.4)	4 (2.0)	10 (3.3)	

^a^ Responses in green are the correct answers given the percentage of participants who gave the response as *n* (%).

When asked about key signs and symptoms, one contact described:

Only about spots, and in extreme cases, amputations, or disabilities […] I seek a lot of information. I have known for a long time that HD was the ancient lepra, which had its own communities reserved for those who had it at the time. That there was no cure. People were deteriorating. I always had this information. Now, knowing, in practice, it was only through him [person affected by HD] that I learned it in practice. (Contact).

In terms of knowledge about the cause of HD, health care workers (69.9%) and CHAs (27.8%) received the highest score in terms of knowledge about the cause of the disease, as described:

The HD bacillus […] it takes five to seven or ten years for it to manifest itself. (CHA interview).

HD is a disease caused by a bacterium that is transmitted through constant contact, you know, by living in the same environment permanently, rather than sporadically. (Professional interview).

Although the professionals demonstrated a better understanding of the cause, it is worth noting that 87 (42.8%) of healthcare workers and 133 (44.0%) of CHAs attributed the disease to hereditary factors.

Another critical point is that 82.7% of the index patient, 72.5% of the close contacts, and 84.3% of the community did not know what caused the disease. Regarding the modes of HD transmission, 464 people (35.4%) from the same subpopulations did not know how it is transmitted. Community members had the lowest level of knowledge about the cause and transmission of HD (70.3%):

[…]in my way of thinking HD… is a disease, but it's not communicable because we lived with it for seven years and never passed it on to anyone … I think HD… I think it's transmitted through a person's blood … that's what I know. (Community member).

The highest proportion of correct answers was found among the subpopulations of CHAs (61.2%, 185) and health workers (48.2%, 98), who reported being infected by “airborne transmission”, although some of them (125, 9.5%) associated HD with contaminated soil and insects.

Whether they understood the mode of transmission or not, it evoked feelings of anxiety and nervousness, as evidenced by the following testimonies from those affected: “I was so anxious with fear because I didn't know much […], or the “normality” as in the statement “I felt normal, as if I had any other disease” (person affected by HD).

A total of 1266 people (96.7%) responded that there was treatment for HD, and 1201 (91.8%) stated that this treatment involved specific drugs. The survey also asked about knowledge of preventive therapies, such as chemoprophylaxis for contacts. Regarding their perception of preventive treatment or chemoprophylaxis, the healthcare workers said: “I didn't know about this, it's new/to me […] I already knew about the tuberculosis one, which we already give here, but not about the HD one*”*. (Nurse).

The sense of anticipation was also evident during the interview: “[…] *[chemoprophylaxis] is necessary to break this chain of transmission. If there is no chemoprophylaxis […], how many people will stop transmitting […]? In another place where I worked […] there was a doctor who mentioned that, by that time, the Ministry of Health […] had proposals to carry out chemoprophylaxis for contacts of patients undergoing treatment for HD. Will it happen soon?*” (Nurse).

Regarding contagiousness, 1161 persons (88.7%) reported that HD is not contagious during treatment:

I only knew that there was a treatment and that there was a cure. The nurse started treatment. They said that after fifteen days of treatment, you can no longer transmit it, and stop transmitting. (Index Patient).

I never stopped to think about it […] because I knew that it was a contagious disease… but that there was a treatment… […] Then, after we had passed, I saw that depending on the severity of HD… It's possible to be treated without incurring disabilities… (Index Patient).

Although people are aware of the treatment, 22.7% believed that the condition would be permanent: “I was afraid of being sick forever, and of passing it on to people, especially my children.*”* (Person affected by HD).

1122 people (85.7%) mentioned disabilities that can be prevented. On this specific issue, those affected scored the lowest at 78.8%.

The mean and 95% confidence intervals for the KAP questionnaire scores were calculated (Table 5). For all subpopulations, this value was 4.84 (95% CI 4.76–4.92). Healthcare workers had the highest mean score (6.35, 95% CI 6.25–6.46), while community members had the lowest (3.75, 95% CI 3.60–3.90).

**TABLE 5:** Mean and range of scores on the applied scales for people approached in Ceará in 2020.

Sub‐populations	KAP measure (17 items) range 0–7		EMIC‐CSS (15‐items) range 0–30		SDS (7‐items) range 0–21		Empowerment scale (25‐items) range 25–100		EMIC‐AP (15‐items) range 0–45	
	Mean (95% CI)	Range	Mean (95% CI)	Range	Mean (95% CI)	Range	Mean (95% CI)	Range	Mean (95% CI)	Range
Index patient	4.03 (3.91–4.16)	2–6			—	—	48.41 (47.46–49.37)	33–65	8.72 (7.73–9.71)	0–34
Close contact	4.67 (4.53–4.81)	1–7	13.26 (12.49–14.03)	0–28	1.44 (1.11–1.76)	0–16	—	—	—	
Community member	3.75 (3.60–3.90)	0–7	14.72 (13.99–15.45)	0–28	2.61 (2.22–3.01)	0–21	—	—	—	
Health care worker	6.35 (6.25–6.46)	4–7	12.90 (9.31–16.49)	6–25	0.40 (−0.39–1.19)	0–4	—	—	—	
Community health agent	5.78 (5.69–5.88)	3–7	16.03 (15.36–16.7)	1–28	0.98 (0.74–1.21)	0–13	—	—	—	

Examining other scales such as the EMIC‐AP, EMIC‐CSS, SDS, and empowerment scales (Table 5), it was found that the EMIC‐AP had a score of 8.72 (95% CI 7.73–9.71), while the empowerment scale showed a score of 48.41 (95% CI 47.46–49.37) for people affected. The highest perceived stigma in the EMIC‐CSS came from CHAs and community members, with scores of 16.03 (95% CI 5.36–16.7) and 14.72 (95% CI 13.99–15.45), respectively. The community had the highest SDS score, 2.61 (95% CI 2.22–3.01), followed by contacts, 1.44 (95% CI 1.11–1.76), and healthcare workers, 0.40 (95% CI 0.39–1.19). Interestingly, CHAs reported a high level of stigma on the EMIC‐CSS scale but had a low score on the SDS scale. Community members also had a high score on the EMIC‐CSS scale but expressed the strongest desire for distancing of all the groups studied.

The mean score achieved on the empowerment scale for those affected was 48.4 (95% CI 47.5–49.4) (Table 5). The factors “Power and Powerlessness” (mean = 18.3) and “Self‐esteem and Self‐efficacy” (mean = 13.7) had the highest empowerment scores (Table 6). It can be seen that people affected in Fortaleza report higher mean scores than those in Sobral for the highlighted factors, except for “Anger or Fair Anger.” Regarding the assessed dimensions, both municipalities scored lower on optimism and future control.

**TABLE 6:** Means and ranges of the Empowerment Scale sum scores, by factors and their scores.

Factors (Empowerment Scale)	Mean sum scores*		
	Fortaleza	Sobral	Both municipalities
**Self‐esteem and self‐efficacy** (9 Items: 4, 5, 8, 11, 13, 16, 17, 21 and 23)	15.3	12.1	13.7
**Power and powerlessness** (7 Items: 6, 7, 9, 14, 15, 19 and 20)	18.5	18.1	18.3
**Community activism** (6 Items: 2, 10, 18, 22, 24 and 25)	9.8	8.1	9.0
**Optimism and Control of the Future** (3 Items: 1, 12, and 24)	5.6	5.1	5.4
**Fair anger or anger** (3 Items: 3, 6, and 9)	10.1	10.9	10.5
**Total score** range 9–36	51.0	45.8	48.4

*Corresponds to the mean of the scale items by municipality and the mean across municipalities.

Multivariate linear regression analysis (Table 7) showed that, overall, women living in Sobral achieved the highest level of knowledge. Conversely, people without religion were associated with a lower level of knowledge.

**TABLE 7:** Multivariate Linear Regression Analysis of KAP score, SDS, EMIC‐AP, EMIC‐CS, and Empowerment, in relation to socio‐demographic and knowledge variables.

Variables*	Regression Coefficient (95% CI)	Standard error	*p*
**KAP (index patient, close contact, community, health care workers and CHA) (R‐squared = 0.0268)**			
Constant	4.59 (4.42 to 4.77)	0.09	< 0.001
City (Sobral)	0.20 (0.06 to 0.34)	0.07	0.005
Gender (Female)	0.36 (0.19 to 0.52)	0.09	< 0.001
Religion (without religion)	−0.32 (−0.48 to −0.15)	0.08	< 0.001
**KAP (health workers and Community Health Agent) (R‐squared = 0.0365)**			
Constant	6.69 (6.35 to 7.02)	0.17	< 0.001
Age	−0.02 (−0.02 to −0.01)	0.00	< 0.001
**KAP (index patient, close contact, Community) (R‐squared = 0.0494)**			
Constant	4.54 (4.24 to 4.85)	0.15	< 0.001
City (Sobral)	0.26 (0.10 to 0.42)	0.08	0.002
Age	−0.02 (−0.02 to −0.01)	0.00	< 0.001
Religion (Others)	0.25 (0.06 to 0.43)	0.10	0.010
**SDS (R‐squared = 0.0758)**			
Constant	3.88 (2.98 to 4.78)	0.46	< 0.001
Religion (without religion)	−0.82 (−1.25 to −0.38)	0.22	< 0.001
Early symptoms (loss of sensation)	−0.46 (−0.84 to −0.08)	0.19	0.019
Hansen's disease is more likely to be permanent (don't know)	0.70 (0.22 to 1.19)	0.25	0.004
KAP Score	−0.45 (−0.63 to −0.27)	0.09	< 0.001
**EMIC‐AP‐ index patients (R‐squared = 0.1253)**			
Constant	13.39 (9.83 to 16.95)	1.82	< 0.001
City (Sobral)	−2.57 (−4.44 to −0.69)	0.96	0.007
Hansen's disease is transmitted by (eating together)	−7.72 (−9.16 to −6.27)	0.74	< 0.001
Age	−0.09 (−0.14 to −0.03)	0.03	0.002
Early symptoms (Others)	4.26 (2.30 to 6.21)	1.00	< 0.001
**EMIC‐CSS (R‐squared = 0.0969)**			
Constant	16.8 (15.94 to 17.65)	0.44	< 0.001
City (Sobral)	−2.81 (−3.62 to −2.01)	0.41	< 0.001
Hansen's disease contagious while on treatment	2.05 (0.80 to 3.30)	0.64	0.001
Cause of Hansen's (don't)	−1.08 (−1.99 to −0.17)	0.46	0.020
Early symptoms (don't know)	−1.92 (−3.29 to −0.55)	0.70	0.006
Hansen's disease caused by (unclean environment)	3.32 (1.88 to 4.75)	0.73	< 0.001
**Empowerment ‐ index patients (R‐squared = 0.2538)**			
Constant	44.7 (41.05 to 48.34)	1.86	< 0.001
City (Sobral)	−4.65 (−6.45 to −2.86)	0.92	< 0.001
Religion (Others)	−2.75 (−4.64 to −0.86)	0.96	0.004
Age	0.13 (0.07 to 0.18)	0.03	< 0.001
EMIC‐AP score	0.18 (0.05 to 0.31)	0.07	0.007
Hansen's disease transmitted by (eating together)	−8.96 (−11.11 to −6.80)	1.10	< 0.001

*The table highlights the variables with the highest level of significance based on the multivariate analysis.

When the regression was applied only to health workers and CHAs, age was associated with lower knowledge levels. Excluding health professionals and CHAs from the analysis revealed that living in Sobral and following non‐Catholic religions were associated with good knowledge, while being older was associated with lower knowledge.

In terms of the desire for social distance, not knowing whether HD is permanent was associated with a greater desire for social distance. In contrast, a high KAP score, not belonging to a religion, and not knowing the early symptoms of HD were associated with a lesser desire for social distance.

Regarding the EMIC‐AP, people living in Sobral, who were older and believed that HD is transmitted by eating together, perceived less stigma, whereas those who knew the early symptoms perceived more.

For the EMIC‐CSS, living in Sobral, and being unaware of the early symptoms and cause of HD were associated with a lower perception of stigma, as well as the belief that the disease is caused by unclean environments and is contagious during treatment, despite a high level of perceived stigma.

Regarding empowerment, living in Sobral and believing that HD is transmitted through shared meals were associated with fewer manifestations of empowerment. Having a high KAP score, belonging to another religion, being older, and not knowing how HD is transmitted were also associated with fewer manifestations of empowerment.

From this perspective, the qualitative analysis highlights relevant aspects, as can be seen in the following statement by a patient, an index, reflecting on the elements considered in the empowerment scale, as well as the verified correlation between a lower perceived stigma and manifestations of empowerment:

I was afraid and wanted to isolate myself from people […], but then every time I went for an appointment, I interviewed the doctor because I wanted to clear up my doubts […], so I received a lot of good guidance about the disease […], but I really wanted to isolate myself from people. Then I became aware […] that I was being treated and was going to be cured. (Index patient).

## 4 Discussion

The present study provides evidence on knowledge, attitudes, and practices regarding HD in Brazil, with a focus on stigma, social distancing, and empowerment. Among the knowledge dimensions, we found that the lowest percentage of correct answers was given for “cause of HD” and “transmission of HD.”

A similar study conducted in India produced different results, with lower percentages of people giving the correct answers regarding the cause (10%) and mode of transmission (2%). These percentages are lower than those found in our study, which indicates a higher level of knowledge in Brazil: 22.5% for the cause and 33.3% for the mode of transmission. Interestingly, health workers, particularly CHAs, continue to primarily associate HD with skin patches rather than with patches accompanied by loss of sensation. However, HD primarily affects peripheral nerves, and nerve thickening is one of the three cardinal signs defined by WHO guidelines. In this study, only 5.6% of CHAs associated HD with neural symptoms such as loss of sensation [29].

In the Brazilian context, this study found higher levels of knowledge than a similar study conducted in Olinda, where the percentages of correct answers for cause, mode of transmission, and symptoms were 15.3%, 9.4%, and 17.0%, respectively [12]. Disseminating accurate information about the cause, transmission, and treatment of HD can play a key role in reducing stigma, promoting early diagnosis, and preventing the spread of the disease. Effective interventions should involve not only health workers, but also community leaders, people affected by HD and their family members, educators, and other local stakeholders [30, 31, 32, 33].

In general, we found that most people knew that the disease could be cured with treatment (97.0%), treated with medication (92.0%), and is not contagious during treatment (89.0%). They also knew that disabilities could be prevented (86.0%). These findings were similar to those from the India study, with 93.0% knowing that the disease is curable with treatment and 97.0% knowing that it can be treated with medication. However, the percentages of people who knew that HD is not contagious during treatment (54%) and that disabilities can be prevented (65%) were lower in India [9].

Interestingly, 23% of people still believed that the disease is permanent, with community members scoring highest on this belief. Similar findings were reported in India, where 26% of people believe HD is permanent, with the highest scores among healthcare workers [9]. This belief may be related to the fact that if an individual presents with a disability caused by HD at the time of diagnosis, this sequela may persist at the time of discharge. Therefore, healthcare professionals must provide clear guidance not only at the time of diagnosis but also throughout the discharge process. Early diagnosis and appropriate follow‐up are essential for preventing physical disabilities [34].

The highest mean knowledge score was found among health workers, including CHAs. This is probably because the HD programme forms part of the Primary Care Facilities programme within the Brazilian public health system [1], meaning that healthcare workers and CHAs have received specific HD training. Incorporating HD into the routine of health programmes and health graduation formation is a key strategy for promoting early diagnosis, preventing HD, and strengthening local capacity through in‐service training. Good knowledge of HD has been shown to predict positive attitudes and adherence to good treatment practices [8]. Other studies have also demonstrated higher levels of knowledge among health workers [9, 10].

Although healthcare workers had a higher knowledge score than the other respondent groups, it is essential to note that many professionals still associated HD with symptoms such as tingling, bleeding, coughing, haemorrhages, blisters, and rashes. It is also noteworthy that, although Community Health Workers work alongside other health workers in the primary care setting, they have less knowledge about the disease and believe that HD is limited to patches without loss of sensation. Some of them thought that HD is associated with an unclean environment. Considering that HD is a treatable peripheral nerve disorder [35], the lack of knowledge among CHAs regarding sensory loss as a characteristic of HD may lead to late diagnosis. Therefore, ongoing educational efforts are needed to improve these health workers' knowledge.

A systematic review showed that healthcare workers often fear treating people with HD and becoming infected. The factors most associated with positive perceptions of the disease were educational level, having received training on HD, having worked in the field for more than five years, being male, and knowing someone with the disease [4]. This also highlights the importance of training.

Community members had a lower level of knowledge about aspects of HD than contacts who already had an understanding of the disease, usually gained through talking to people with HD or discussing the disease with healthcare workers. These results are consistent with those reported by Jiménez et al. in another Brazilian endemic area, where community members and contacts also had limited knowledge of HD. They noted that most of their knowledge came from healthcare workers [12].

An exploratory study conducted in Brazil suggests that people with HD who feared community isolation and social exclusion were ten times more likely to wait a long time before seeking medical help for their symptoms [16]. The study also found that lower knowledge was associated with an increased desire for social distance from people affected by HD among community members, contacts, and health workers. Social distance may be interpreted as a proxy for fear. If greater knowledge reduces fear, then increasing community knowledge may also reduce the delay in seeking treatment [36].

In the multivariate analysis, not having religion was associated with low knowledge. Although studies conducted in Brazil have addressed the influence of religion on patients' experiences, few articles have been found that associate religiosity with knowledge about HD. While these studies do not establish a direct relationship between religion and better knowledge of the disease, they highlight that religious beliefs can influence treatment adherence—either as a source of support or, in some cases, as a barrier—depending on how patients experience their faith [37, 38].

In this study, CHAs obtained the highest mean EMIC‐CSS scores (16.0), indicating the level of stigma towards people with HD. On the other hand, the SDS scores (0.98) were lower than those of other subpopulations. One explanation for this finding is social desirability bias: as health professionals living within the community, CHAs may have responded in ways they believed would be better received, considered more correct, or viewed more favourably. This is because the EMIC scale often prompts respondents to consider what people in their community think or do, enabling them to express stigma more freely by projecting it onto others. However, when asked directly about their own attitudes, as in the SDS scale, they may fear judgment or repercussions for their speech [39, 40].

The level of perceived stigma in the community (14.7) was similar to that found in studies conducted in India (16.2) [9] and Indonesia (17.0) [10]. It is important to note that the community had limited knowledge, which can directly affect negative attitudes towards the disease [41]. This may explain why the community showed the greatest desire to distance itself from the people affected by HD. This aligns with the findings of a study conducted in India, in which community members expressed a desire to maintain social distance and reported avoiding physical contact, social interaction, and sharing meals with people affected by the disease [36].

In this study, the highest mean social distance scores were found among community members (2.6) and close contacts (1.4). These scores were much lower than those found in India and Indonesia. In India, the mean social distance score was 8.2 for community members and 7.0 for close contacts [9], while in Indonesia it was 8.6 for community members and 9.1 for close contacts [10]. It is worth highlighting that people who did not know whether HD was permanent had a stronger desire for social distancing. A study conducted in Chandauli, India, showed that increased HD‐specific knowledge was associated with significantly lower SDS scores [19].

With an EMIC‐AP score of 8.72, participants reported a significant level of perceived social stigma. Previous studies have observed the influence of socio‐educational factors and restricted participation. Implementing educational and training programmes aimed at reducing stigma has proved essential in combating internalised stigma and promoting the social inclusion of people affected by HD [31, 42].

The multivariate analysis revealed that in Sobral, there was greater knowledge of HD, less community‐based stigma, and a lower perception of stigma among those affected. These findings can be attributed to a set of interconnected factors reflecting local public policies and the city's social characteristics [43]. Notably, the municipality of Sobral has a well‐structured primary healthcare system with 100% coverage. From a community perspective, the city exhibits characteristics typical of medium‐sized Brazilian towns, where interpersonal relationships are closer and daily interactions foster mutual understanding. In light of these insights, further exploration is recommended into how community dynamics, including proximity between neighbours and mutual knowledge, influence the perception of HD‐related stigma and how this contributes to reducing it.

The Rogers' Empowerment Scale is one of the most widely used [44]. While the linear regression analysis in Sobral revealed an association between Sobral and lower stigma and greater knowledge, participants from Fortaleza reported higher levels of empowerment in nearly all assessed domains, achieving an overall score of 51.0 in Fortaleza compared to 45.8 in Sobral. These findings suggest that aspects related to empowerment are present in both locations, including cultural and demographic factors, gender, income, and education, and should be further explored [23].

The highest score across both municipalities was found in the ‘Power and Powerlessness’ domain, indicating a greater awareness of power dynamics in participants' lives. Even if stigma is present in these areas, which are often linked to prejudice, discrimination, and social exclusion, and it can negatively affect self‐image and psychological well‐being [44, 45, 46]. One possible explanation for this finding is that some people may develop resilience mechanisms in response to stigma and discrimination. These mechanisms can include becoming aware of their rights, seeking social support, and taking action against discrimination [47].

Therefore, perceived stigma may be associated with higher or lower levels of empowerment, depending on how individuals cope with adversity and on whether those persons affected have access to interventions that address coping mechanisms. The relationship between stigma and empowerment is always complex. Increased empowerment can also lead to reduced stigma [48].

A study was conducted in Brazil to understand better how HD affects people's lives. Patients undergoing treatment were interviewed, and 94% of participants mentioned stigma and prejudice. A proportion of 40% reported feeling depressed and poor, sad, and 69% experienced problems at work after diagnosis. Of all those interviewed, 45% rated their quality of life as poor to very poor [47]. HD continues to affect both endemic and non‐endemic countries, negatively impacting individuals' lives in terms of mobility, interpersonal relationships, marriage, employment, leisure, and social and religious participation [49, 50].

This study provides essential support for one of the objectives of the national and global HD strategies, which focuses on strengthening information, communication, and educational activities on HD, involving people affected by the disease, their families, and communities. In line with the study's findings, the PEP++ programme considers it crucial to disseminate contextualised information that strengthens local knowledge, addresses specific misconceptions and beliefs, and combats stigma, prejudice, and discrimination [9]. It is expected that this will lead to improved treatment‐seeking behaviour, increased uptake of chemoprophylaxis among contacts, and eventually interruption of transmission.

## 5 Limitations

The cross‐sectional design of the study in both municipalities limits our ability to understand cause‐and‐effect relationships. However, the breadth of the study, the diversity of subpopulations and settings, and the use of a mixed‐methods design validate the findings.

## 6 Conclusions

This study revealed a significant lack of knowledge, as well as specific beliefs and misconceptions regarding key aspects of HD—among both the community and health workers. These gaps were associated with high levels of perceived negative attitudes and behaviors towards HD as well as a greater desire to distance oneself socially from affected individuals. These findings highlight the critical importance of context‐specific health education strategies that address knowledge deficits, challenge misconceptions, and reduce stigma and prejudice.

## Conflicts of Interest

The authors declare no conflicts of interest.
